# Regional anesthesia for pain control in children with solid tumors—a review of case reports

**DOI:** 10.3389/fped.2023.1275531

**Published:** 2024-01-11

**Authors:** Aliya Baizhanova, Azhar Zhailauova, Vitaliy Sazonov

**Affiliations:** ^1^ School of Medicine, Nazarbayev University, Astana, Kazakhstan; ^2^ Department of Surgery, School of Medicine, Nazarbayev University, Astana, Kazakhstan; ^3^ Pediatric Anesthesiology and Intensive Care Unit, National Research Center for Maternal and Child Health, University Medical Center, Astana, Kazakhstan

**Keywords:** regional anesthesia, oncology, pediatric, pain, palliative care

## Abstract

**Introduction:**

Around seventy percent of all childhood cancer patients suffer from severe pain. This pain can arise from various sources, including tumors themselves, pain caused by metastasizing tumor cells or as the outcome of therapy meant to deal with tumors. If managed inadequately, such pain can lead to many hazardous sequelae. However, there are extreme cases when pain does not respond to standard treatment. For such cases, regional anesthesia or nerve blocks are utilized as the utmost pain control measure. Blocks are used to treat pain in patients who no longer respond to conventional opioid-based treatment or whose worsened condition makes it impossible to receive any other therapy. The data regarding the use of regional anesthesia for such cases in the children population is limited.

**Methods:**

For this review we searched for case reports in Scopus and PubMed from inception to 2023. The descriptive search items included terms related to childhood cancer and the description of each block. The inclusion criteria for review include children (0-18 years old) receiving oncology-related surgical procedures or palliative care. The data collection was limited to solid tumor-related cases only. We analyzed a total of 38 studies that included case reports and one retrospective study.

**Results and discussion:**

It was concluded that nerve blocks, although rarely performed, are a safe and efficient way of pain control in children with solid tumors. The major settings for block performance are postoperative pain control and palliative care. We observed that block indication and its outcomes depend on unique health circumstances in which they should be performed. Patients with similar diagnoses had differing outcomes while receiving the same block treatment.

## 1 Introduction

### 1.1 Pain in cancer patients

Although painful sensations are common in many health disorders, persistent severe pain in cancer patients is a sign of cancer advancement into nearby tissues (1). In cancer patients, pain often originates from a tumor, with growing cells causing inflammation and destroying nearby cells, or as an outcome of surgical treatment meant to deal with tumor cells. During the process of treatment, most patients are exposed to intense multimodal therapies, including diverse invasive procedures, surgery, chemotherapy, and radiation (2). To some extent, all above mentioned therapies result in pain, with the tumor resection surgeries estimated to be the most painful (3). If not treated promptly, postoperative pain can have significant sequelae on physical and mental health (4). According to systematic review and meta-analysis performed on patients after thoracotomy and breast cancer surgery, timely applied regional anesthesia prevented occurrence of chronic postoperative pain in twenty—twenty five percent of the cases (5). Poorly ameliorated pain puts one at risk of developing hypoventilation due to reduced vital capacity, myocardial infarction due to coronary ischemia and urinary retention (6). Analyzing the results from a US national survey (4), it was deduced that if pain endurance exceeds its expected healing time, patients are prone to develop mental distress, anxiety, and hesitancy to participate in treatment-related procedures.

### 1.2 Cancer pain in children

Globally, it is estimated that about one in five hundred children develops cancer in their lifetime, with seventy percent of childhood cancer patients suffering from severe pain (7). Especially during the end of life period, children suffer from enormous refractory pain (8). Poor pain control mainly causes significant concern in pediatric patients, a group whose capacity is thought to be insufficient to communicate regarding pain severity and make adequate analgesia requests (9). Inadequate pain management within pediatric patients originates from inability to assess pain accurately. Two major encountered barriers are the subjective nature of pain and avoidance behavior (10). Although standardized pain assessment tools are able to define pain strength, they fail to grasp and accurately deliver actual painful sensations the patient may feel. As for the latter, there are cases in practice describing patients and their parents intentionally hiding pain from medical staff due to the anxiety and fear of being hurt by medical procedures (11).

### 1.3 Regional anesthesia and cancer pain in children

Conventional method of pain management consists of application of sequential amplification of analgesic therapy that was recently updated for adults and adolescences and includes NSAIDs as a first line therapy, opioids as the second line therapy, and addition of adjuvants such as ketamine, lidocaine and gabapentinoids as the third line therapy (12, 13). Regardless of the given considerations, during the cases of refractory pain in cancer patients, when opioid regimens result in ineffective treatment with subsequent complications such as respiratory depression, nausea and vomiting, constipation and opioid tolerance (13), regional anesthesia could be considered as a powerful alternative (14). Regional Anesthesia (RA) in pediatrics became popular only several decades ago (15). Application of RA among children was documented in several studies conducted by French-Language Society of Paediatric Anaesthesiologists (16) and by Pediatric Regional Anesthesia Network (17) supporting safety of the method under general anesthesia. Important outcomes of RA use are its effect on reducing opioid consumption, episodes of respiratory depression, and pain intensity subsequently (18).

Regional anesthesia techniques, consisting of neuroaxial and peripheral neural blocks (PNBs), are less utilized in pediatric patients due to a lack of understanding of the risks associated with such procedures (19), although association with ultrasound guidance made application of RA safer (15). Owing to a broad range in pediatric patients, spanning from neonates to adolescents, the anatomy of injection sites and administered doses exhibit significant variability (20). Anatomic variability exposes anesthesiologists to additional challenges as it influences the precision of block performance which, in turn, affects the intensity and extent of analgesia (20). This requires more skilled personnel to administer the block, which might as well contribute to a lesser practice of block performance in children (21). Complications from placing regional blocks may include nerve damage, local anesthetic systemic toxicity, vascular puncture (22), catheter dislodgement and infection (23). However, there is a low incidence of complications corresponding to 2.4 cases per 10,000 associated with neurological complications and 14 cases out of 10,000 related to respiratory depression (17).

This review discusses the types of regional anesthesia techniques and conditions that affect selection of a certain block based on the case report review. By doing so, we aim to build a general overview of the block administration efficacy and outcomes of each block's performance. Since regional anesthesia is rare among children, with all case information being scattered, this summarized overview can be used as an advisory when dealing with severe cases. The secondary aim is to look into the application of regional anesthesia for pediatric oncology patients during the end-of-life period. We expect that block indications are case-dependent, and the choice of the block is greatly affected by the individual's clinical scenario, as patients with the same diagnosis may receive differing block preferences.

## 2 Materials and methods

**Figure 1:**
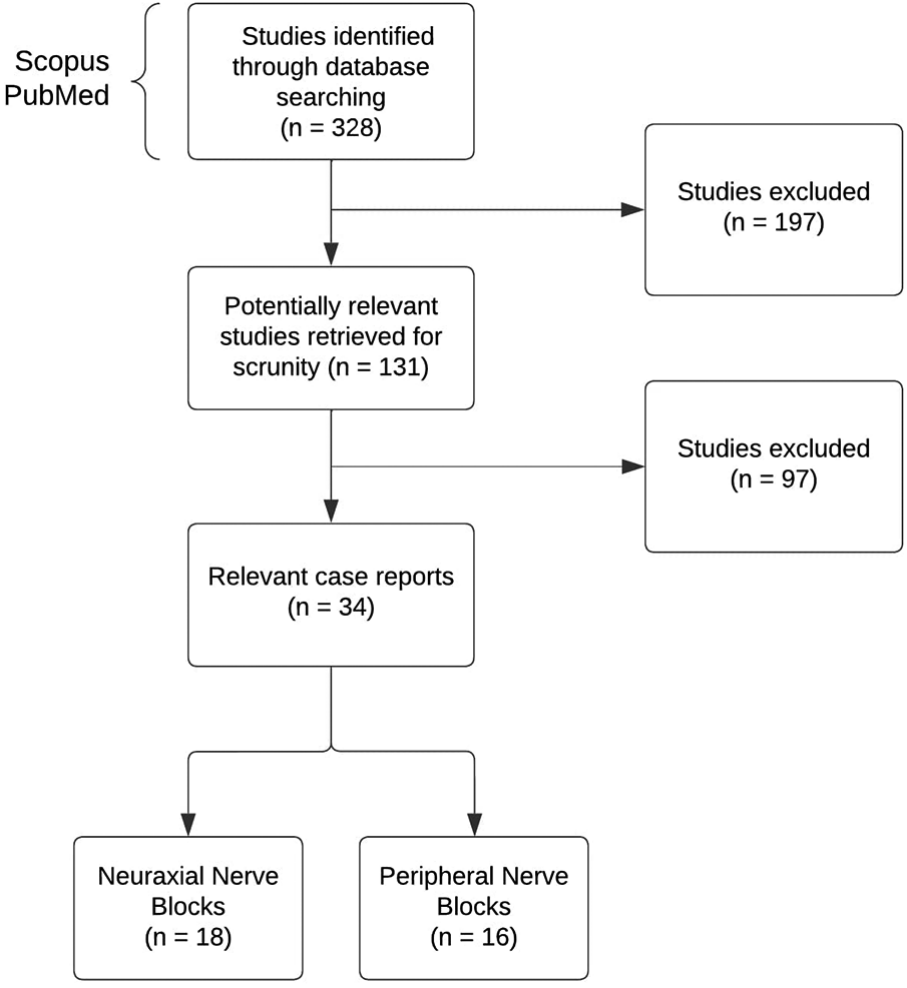
The search and exclusion strategy.

We searched for case reports in Scopus and PubMed from inception to 2023. The descriptive search items included terms related to childhood cancer and the description of each block. The key terms for the search and the amount of found literature are shown in Table 1. Several articles were also located through a reference list of relevant literature, with cases citing previous cases as a guidance. The inclusion criteria for review include children (0–18 years old) receiving oncology-related surgical procedures or palliative care. The data collection was limited to solid tumor-related cases only. Figure 1 illustrates the search and exclusion strategy.

**Table 1:** Search strategies.

Key terms for search	Results from March 15, 2023
Database: Scopus	
Childhood cancer	64,964
Cancer pain	167,749
Neuraxial block	1,715
Peripheral nerve block	12,027
Pediatric palliative care	47,538
Postoperative analgesia	200,958
Nerve blocks in children	3,387
Intrathecal analgesia	8,516
Regional anesthesia	210,300
Erector spinae plane block	669
Epidural analgesia	25,946
Database: PubMed	
Childhood cancer	54,738
Cancer pain	75,597
Neuraxial block	1,254
Peripheral nerve block	27,350
Pediatric palliative care	6,897
Postoperative analgesia	30,850
Nerve blocks in children	3,014
Intrathecal analgesia	3,957
Regional anesthesia	85,170
Erector spinae plane block	1,195

Since blocks in pediatric oncology patients are rare, only a few case reports were available. We analyzed a total of 38 studies that included case reports and one retrospective study. Three cases were excluded from the analysis since they were written over 30 years ago. This study aims to do the relevant review, so all cases dating from the 2000s were included. Although the above-mentioned cases were excluded from the analysis, they were kept for discussion as they describe the first instances of pediatric block performances. Of these 34 studies, 6 were case series, with the remaining part constituting case reports. Sixteen cases were for peripheral nerve blocks and 18 for neuraxial nerve blocks.

## 3 Results

Cases included in this review described the following procedures: peripheral nerve blocks, intrathecal infusions, neurolysis, epidural infusions, plexus, and ganglion blocks. The summary of blocks and patients’ characteristics is presented in Table 2. It is important to note that some cases had no available information on the age or weight of the patients. Despite such a gap, we faced no issues related to the data analysis as the other relevant details were sufficient to create a descriptive table. The detailed description of each case and the outcome of each procedure is summed up in Table 3.

**Table 2:** Patient and block characteristics from studied case reports.

Patients	Blocks (*n* = 34)	Regional Anesthesia techniques (*n* = 34)	
Age, years (*n* = 30)	11 (6–15)^a^	Neuraxial blocks	18
Sex (*n* = 23)		Single injection	4
Female	13	Catheter infusions	9
Male	10	Pump	5
Weight, kg (*n* = 13)	23 (15.6–41)^a^	Peripheral nerve blocks	16
Local anesthetic type		Single injection	5
Bupivacaine	22	Continuous	11
Ropivacaine	10		
Lidocaine	1	Epidural	9
Mepivacaine	2	Single injection	-
Ethanol	2	Continuous	9
Phenol	3	Intrathecal	5
Guidance used in block placement		Catheter	1
Ultrasound	4	Pump	4
Fluoroscopy	5	Peripheral Nerve Blocks	16
Nerve stimulator	1	Celiac Plexus	3
Ultrasound with a nerve stimulator	7	Brachial Plexus	7
CT	3	Impar ganglion	1
Unassisted	14	Femoral	3
		Sciatic	1
		Lumbar plexus	1
			
		Erector Spinae Plane Block	3

^a^ Range is given with mean + IQR.

**Table 3:** Case reports included in this study.

Author, year	Age, years	Gender	Weight, kg	Description/diagnosis	Block technique	Outcomes
Munoz et al. (24)^a^	7	Male	N/A	Tumor of the 11th right rib	Erector spinae plane block	– Pain score of 11/30, according to the COMFORT Behavior scale – No opioids until hospital discharge on the 3rd postoperative day
Balaban et al. (25)^a^	6	Male	25	Femur fracture	Lumbar erector spinae plane block	– 30 min postoperatively, Wong-Baker Face scale pain score of 0 – No opioids until day 12th postoperatively
Grap et al. (26)	16	Female	51	Synovial sarcoma	Femoral and sciatic epineural catheters	– Pain score decreased to 5/10 within the first 24 h – All catheters were removed on day 12 – Patient was discharged home 3 days later with pain score of 3/10 – Patient was still receiving adjuvant opioids to relieve pain 1 year after surgery – No opioid requirement on year 2 postsurgically
Maruta et al. (27)^a^	6	Male	21	Bilateral hip subluxation	Continuous epidural anesthesia	– No pain complaints after surgery – No neurological complications – Recovered muscle power
Bozkurt et al. (28)	2	Female	N/A	Pelvic/sacrococcygeal mass	Epidural catheter	– Good pain relief after block – Patient was transferred to another hospital for chemotherapy after 2nd week – No monitoring was initiated after that
Aréchiga-Ornelas et al. (29)	**5**	Male	15.6	Abdominal neuroblatoma	Bilateral erector spinae plane block Caudal block	– The opioid dosage was reduced by 75% over the next 36 h – 75% pain decrease by Wong-Baker Face scale – 100% pain decrease by FLACC – Improved alertness – No block-related complications
Restrepo-Garces et al. (30)	3	Male	N/A	Refractory perineal-cancer-related pain. Embryonal bladder/prostate rhabdomyosarcoma	Neurolytic injection on the impar ganglion	– Pain decreased by 70%, according to FLACC – Improvement in sleep pattern – Due to tumor invasion, the patient developed malignant intestinal obstruction and progressive respiratory depression – Patient died 5 days post block
Santana (31)	12	Female	N/A	Metastatic rhabdomyosarcoma	Tunneled epidural catheter	– Patient had reported an analgesic effect – Opioid dosage was reduced – Catheter leakage and bleeding – Bleeding resolved after removal of the catheter
Mele (32)	16	Female	N/A	"Ewing-like” sarcoma	Intrathecal pump	– Patient was able to participate in physical activities – Adequate pain control with fewer side effects – Due to tumor progression, an increased dose of opioids was administered – Patient was transferred to palliative care
Conn et al. (33)	16	Male	N/A	Osteosarcoma	Intrathecal drug delivery (IDD)	– Improved quality of life – IDD helped to avoid opioid tolerance and lowered side effects
	15	Female	N/A	Osteosarcoma		
Bentley et al. (34)	16	Female	N/A	Melanoma and metastases to the small intestine	Intrathecal pump	– VAS pain before block: 9 – VAS pain at 3 months follow up: 7
Bengali et al. (35)	15	Female	N/A	Metastatic squamous cell cancer of the anus + combined immune deficiency syndrome	Intrathecal pump	– Patient had her vivid dreams, urinary retention, and kidney markers improved – Patient had better subjective pain scores
Higuchi et al. (36)	14	Female	N/A	Neurofibromatosis type I Malignant peripheral nerve sheath tumor	Intrathecal PUMP	– Patient was alert and could enjoy reading, eating, and watching TV – Patient had her bowel movements resumed – Patient required fewer opioids for pain control
Whyte & Lauder (37)	3	Male	14	Advanced pelvic rhabdomyosarcoma	Intrathecal infusion	– Pain relief with less hypotension and sedation – Patient was able to be transferred to a home for 2 days with no pain – Patient died 2 weeks later due to tumor progression
Tashiro et al. (38)	10	Female	20	Right ilium osteosarcoma	Intrathecal neurolytic block	– Patient reported decreased touch and pain sensation without motor block – Discharged 2 weeks after block – No complications related to block – Patient was able to go outside using a wheelchair – Significant increase in the quality of life. Patient was able to spend time with family without severe pain
Anghelescu et al. (39)	16	N/A	N/A	Clear cell sarcoma	Epidural catheter	– no change in the mean score of pain
	6	N/A	N/A	Neuroblastoma	Epidural catheter	– no change in the mean score of pain – decrease in opioid requirement
	18	N/A	N/A	Rhabdomyosarcoma	Epidural catheter	– decrease in the mean score of pain – decrease in opioid requirement
	4	N/A	N/A	Neuroblastoma	Epidural catheter	– decrease in the mean score of pain – decrease in opioid requirement
	18	N/A	N/A	Osteosarcoma	Epidural catheter	– decrease in opioid requirement
	8	N/A	N/A	Rhabdomyosarcoma	Epidural catheter	– decrease in the mean score of pain – decrease in opioid requirement
Burgoyne et al. (40)	14	Male	N/A	Osteosarcoma	Femoral catheter	– Patient was discharged from hospital – Catheter was maintained in the outpatient settings for 26 of 41 days – Reduced opioid consumption – Patient underwent tumor resection and received femoral prosthesis 2 months later
	13	Female	N/A	Osteosarcoma	Femoral catheter	– On day 33, a catheter was removed due to catheter obstruction and replaced with another – Continuous nerve block was stopped on day 81 due to minimal pain – Reduced opioid consumption – The patient underwent left hip disarticulation 5 days after
	8	Female	N/A	Osteosarcoma	Brachial plexus	– Discharged from the hospital a week later – Catheter removal on day 36 – Reduced opioid consumption
Kaddoum et al. (41)^a^	Elementary school child (7–10)	N/A	35	Osteosarcoma	Brachial plexus, nerve sheath catheter	– No complaints of phantom limb pain (PLP) from day 1 to day 3 postoperatively – Mild pain on day 4 as 3/10 according to NRS – Patient reported no pain during week 3 – 5 months later, the patient died of lung metastases
	Elementary school child (7–10)	N/A	41	Osteosarcoma		– Postoperatively, the patient complained of right arm PLP and back pain rated at 6/10 on the NRS – Severe PLP on day 7 – No pain on day 9, hence catheter was removed – Patient complained about “constant phantom pain” on day 16, requiring an increased dose of methadone and gabapentin – No pain complaints on day 71 postoperatively. All medications were discontinued
	Elementary school child (7–10)	N/A	45	Osteosarcoma		– Patient reported no pain postoperatively – No morphine use during the next 24 h – All catheters were removed on day14 postoperatively – On day 26, all pain medications were discontinued – There were no complaints of PLP in the 18 months of follow-up
	Elementary school child (7–10)	N/A	56	Osteosarcoma		– Postoperatively, the patient was receiving multimodal analgesia until day 7 and reported no PLP – Pain 10/10 on day 8. The patient received morphine – After this, the patient had no pain complaints – All catheters were removed on day 14 – No PLP complains after that – Patient died of progressive lung metastases 1 year after block
Anghelescu et al. (42)	14	Male	34.8	Hepatoblastoma	Celiac plexus block (CPB)	– Immediate pain relief with a pain score of 0 – Patient has been weaned off opioid pain medications 1 week after CPB – Patient has remained off scheduled opioids for 6 months – This patient received a liver transplant and survived
	8	Female	18.3	Rhabdomyosarcoma	Celiac plexus block (CPB)	– Decreased pain score in the 1st week – Patient received fewer opioid during week 2 – Disease progression outside the abdomen caused opioids to be given at a higher dose, with the subsequent sharp increase in dose at the end of death
Shimazaki et al. (43)	13	Male	N/A	Osteosarcoma	Intrathecal neurolytic block	– Immediate pain relief – Decreased opioid use – Patient was able to go back home and enjoy school activities – Patient experienced no pain until his death
Tognu et al. (44)^a^	2	Female	7	Ewing family tumor	Continuous lumbar plexus	– Immediate pain relief (ChIPPS <4) – Patient slept well at night following surgery – No additional morphine was required – No nausea and vomiting were observed
Kumar et al. (45)^a^	7 months	N/A	10	Rhabdomyosarcoma	Supraclavicular brachial plexus	– Patient has reported no pain postoperatively – On day 4, slight subcutaneous emphysema was noted over the right-mid axillary area – x-ray demonstrated a slight tracheal shift to the left side, which was ruled out to be a sign of pneumothorax development – Pneumothorax was most likely caused by the puncture of the pleura

^a^ Patients received a block for pain management for a surgical procedure or for early postoperative period.

### 3.1 Peripheral nerve blocks

Peripheral neural blocks (PNBs) allow the delivery of anesthetic along or close to the specific peripheral nerve of the target body part (46). The data from reports prove the efficiency and versatility of peripheral nerve blocks. The case series reporting osteosarcoma in three pediatric patients have shown excellent efficiency of continuous celiac plexus block in alleviating pain (40). Two out of three children patients received an infusion of local anesthetic through the femoral catheter. All three patients reported reduced pain and had their opioid consumption reduced for several weeks. These case series show successful catheter maintenance in an outpatient for 40 days. Almost all catheter-related cases were done via subcutaneous tunneling. Doing so creates distance between the insertion site and the target peripheral nerve, lowering the possibility of infection from skin bacteria near the insertion site (47). Since most cancer patients undergo chemotherapy as a part of their treatment and have some degree of immunosuppression, infections pose severe risks for all cancer patients. No infection-related complications occurred due to the proper antibiotic catheter management as a prophylaxis measure.

The case of a 7-month-old child undergoing rhabdomyosarcoma-related tumor excision who received supraclavicular brachial plexus block shows that infants are more susceptible to complications of block performance. While the block efficiently alleviated postoperative pain, the patient developed subcutaneous emphysema with no painful sensation (45). An x-ray showed a tracheal shift, further considered a sign of developing pneumothorax. The authors concluded that pneumothorax was most likely caused by an anatomical difficulty in accessing the plexus of more minor children. Although ultrasound (US) guidance was used, it was reported that while making an insertion, no needle tip was seen in some instances, which might have resulted in a pleura puncture. This case shows how seemingly insignificant deviation affects the block performance in patients with different ages and anatomy.

Peripheral nerve blocks are also widely used in treating postoperative phantom pain. Around 80% of cancer-related amputation patients experience phantom pain for at least one year following surgery (40). Four patients with osteosarcoma-related fractures were treated with continuous brachial plexus blocks with 0.25%−0.5% bupivacaine and 0.2% ropivacaine after amputation. It was interesting to observe that despite having no pain complaints postsurgically, all patients experienced mild pain—rated 6/10 and higher—within the first week after the block. In such cases, the opioid requirement was raised again to treat the pain. Some catheters were removed two weeks after the surgery due to reasonable pain control. None of the surviving patients reported pain during the following year's follow-up. In another case, the phantom pain of a 16-year-old female patient with synovial sarcoma, who underwent right hip disarticulation, was treated using sciatic and femoral epineural catheters (26). The patient received adjuvant opioids for one year, and all medications were discontinued by the second year.

The cases describing the usage of block in treating pain after disarticulation procedures show that the sole use of a block might not be enough to alleviate pain completely. The medications and procedures done as a part of multimodal analgesia in conjunction with block analgesia yield better pain control in patients with phantom limb pain. After block, all patients were reported to receive adjuvant medications with lesser opioid burden.

One cannot underestimate the importance of psychotherapy after amputation surgery as a part of postoperative pain treatment. Patients experience severe depression, despair, anxiety, and self-stigmatization after disarticulation surgery (48). The loss of a body part in children not only interferes with the child's daily abilities but also with their social functioning (49). The fear of being left out and not accepted by peers causes significant distress in growing children. According to the authors, if psychological issues are not addressed on time, the distress will lead to the child's inability to adapt to the environment and subsequent poor quality of life. Hence, a great emphasis should be made on a multidisciplinary way of treating pain: alleviating the cause of pain and helping a child navigate such a life-changing procedure.

### 3.2 Neuraxial nerve blocks

#### 3.2.1 Intrathecal blocks

Intrathecal (IT) blocks are performed by inserting a needle into the subarachnoid space and injecting the local anesthetic into the cerebrospinal fluid. An intrathecal analgesia can be successfully provided through a single injection, a continuous flow through a catheter, or a specifically designed pump with a medication reservoir. Such versatility allows pain control in an outpatient setting, letting the patient return to daily life activities comfortably. Intrathecal infusions require less medication to be administered and have 100-fold potency compared to oral or intravenous drug delivery (34). Such attributes of IT reduce opioid-related complications such as nausea, constipation, and delusion (50).

The case report analysis has shown that the decision for the intrathecal block in children's patients is made when they no longer respond to the increasing opioid prescription, suffer from medication-related complications, and are disadvantaged from sedation. A 14-year-old female patient with neurofibromatosis I was treated with continuous infusion of bupivacaine through the subarachnoid catheter. The patient had her bowel movement resumed, and her life quality improved significantly (36). Opioid tolerance, frequently occurring in terminally ill children, is also avoidable with intrathecal blocks. Conn et al. (33) report that intrathecal drug delivery helped to minimize the long-term toxicity caused by high doses of opioids in patients with osteosarcoma who no longer responded to opioids.

IT blocks also proved their effectiveness in pain control of those with intractable pain and whose life expectancy is less than a year (51). The case of a 15-year-old girl with metastatic squamous cell cancer of the anus with combined immune deficiency syndrome has shown that intrathecal pumps significantly improved the patient's last six months of life (35). Such implantation has allowed the patient to participate in life activities such as returning to a limited time in school and spending holidays with her family, something that was previously unattainable due to pain. Nevertheless, an analysis showed that the implantation of IT pumps in younger children is limited due to the pumps’ unavailable medication reservoir size. For now, this problem is solved by using external pumps or port-a-caths, which are found to be confining for patients (35). If designed considering this, palliative pain control in more minor children would be more feasible.

Intrathecal neurolytic blocks, that is, injecting alcohol to disrupt nerves and achieve pain relief, were also described to be a suitable pain control tool for terminally ill patients. A 10-year-old girl diagnosed with recurrent right ilium osteosarcoma received intrathecal neurolytic block as a part of her palliative care (38). Before the block, she was receiving a significant dose of intravenous oxycodone at 1,320 mg/day, equivalent to 2,640 mg/day of oral morphine, which resulted in severe delirium. Once block was performed, the patient reported reduced touch and pain sensations without a motor block. No side effects of opioid treatment were present, and the patient was able to go outside with a wheelchair and spend quality time with family.

#### 3.2.2 Epidural blocks

Epidural anesthesia is achieved by injecting a local anesthetic into the epidural space by inserting a needle between two vertebrae in the cervical, thoracic, or lumbar spine (52). Such block type can be placed at any level of the vertebral column, depending on the desired anesthetic coverage area. Whether performed as a single shot injection or catheter, epidural blocks are extensively used to treat lower extremity, lower abdominal, and urological-related pain (53). The catheters for continuous infusions are tunneled close to the insertion site to avoid catheter dislodgement-associated complications (54). Little is known about using continuous nerve blocks (CNB) at the end of life. The existing evidence show that considerable number of patients who received CNBs experienced significant reduction in mean pain scores as well as decrease in opioid requirements (55). Case series analysis done by Anghelescu and colleagues (42) has shown the use of epidural catheters as a pain control in patients suffering from rhabdomyosarcoma, neuroblastoma, osteosarcoma, and other types of solid mass tumors. The case series demonstrates that the continuous infusion of local anesthetic through epidural catheters significantly reduces pain scores and IV opioid requirements in palliative care patients. Patients were described as “comfortable at the time of death and to not suffer from pain”, proving the efficiency of epidural analgesia in treating cancer-associated pain at end-of-life treatment. It was also observed that tunneled epidural catheters could function for as long as two hundred forty days in pediatric patients without severe complications (42). The lesser opioid requirements have reduced somnolence and allowed better interaction with family members. This benefit is crucial for parents as seeing children being less in pain eases parents’ sufferings associated with such stressful circumstances (56).

There is a big concern about using regional anesthesia in toddlers and preschoolers due to local anesthetic toxicity caused by local anesthetic's damaging effect on the demyelinated axons (57). No case report was found on the neuraxial block used for neonates. The case report of patients with Pelizaeus-Merzbacher disease (PMD), a rare genetic disorder related to lack of myelin production and subsequent white matter abnormalities, describes using an epidural catheter in treating postoperative pain (27). A 6-year-old male patient undergoing PMD-related hip subluxation was treated with a continuous infusion of 0.2% ropivacaine for 46 h. The patient was reported to regain muscle power and was transferred to postoperative rehabilitation five days after block performance. According to Maruta (27), there is no restriction to using regional anesthesia in patients with PMD: the block decision should be made by considering possible complications, such as respiratory failure caused by spasticity for PMD patients. This case report demonstrates a few important considerations that should be resolved beforehand: risk and benefit analysis of block performance and readiness to deal with complications. The patient could have been subjected to more exacerbated neurologic symptoms without further treatment prospects. This study shows that the choice for using regional anesthesia in patients with existing neurologic disorders should be worked out to avoid complications.

It also shows that the extent of the complications after specific procedures greatly varies based on health scenarios. While epidural catheters helped to alleviate postoperative pain in the above-mentioned case, tunneling epidural catheters in patients with metastatic rhabdomyosarcoma have ended unsuccessfully. A 12-year-old female patient with advanced metastatic rhabdomyosarcoma received epidural analgesia for palliative care (31). The catheter was placed without complications, and the patient reported an analgesic effect. However, on day 2, the patient developed massive bleeding and subsequent clogging around the tunnel site. The bleeding stopped once the catheter was removed. Bleeding, although rare, is one of the major complications of catheter placement. According to Santana (31), the tissues of cancer patients are more friable and hence easily prone to traumatic injury caused by catheter placement. For that reason, such procedures should be carried out with great care.

### 3.3 Other blocks

#### 3.3.1 Erector spinae plane block

Although it cannot be fully classified as a peripheral nerve block *per se*, erector spinae plane block (ESP) is an emerging and promising technique for postoperative anesthesia. The main feature of this block involves the injection of local anesthetic in the plane superficial to the erector spinae muscle, targeting the dorsal and ventral branches of the spinal nerves (58). Performed under US guidance, the mechanism of the way block affects spinal nerves is still debated. The existing studies support that ESP can efficiently block anterior, posterior, and lateral thoracic and abdominal walls (59). The main benefit of ESP includes easily recognizable sites of injection as well as the limited risk associated with needle-related injuries (58). Indeed, the case analysis of a 7-year-old male patient with a tumor of the 11th rib has shown that an injection site is distant from neuraxis, pleura, and vascular structures, which minimizes risks of accidental needle-caused rupture (24). Moreover, it was ruled out that local anesthetic delivery through a single injection was enough to provide comprehensive analgesia coverage (25). As postoperative pain control, a 6-year-old male patient received a lumbar erector spinae block for a femur fracture (25). Thirty minutes postoperatively, the patient had 0 scored pain according to Wong-Baker FACES score and received no opioids for 12 days postoperatively.

The continuous infusion of local anesthetic through catheters under bilateral ESP block successfully treated uncontrolled pain caused by abdominal neuroblastoma of a 5-year-old male patient (29). The patient was reportedly receiving multiple rescue doses of morphine daily dose equivalency of 17.3 mg per day, which has led to hypoactive delirium and severe constipation. Postoperatively, the patient had improved alertness, and his opioid dose was reduced for the next 36 h. However, due to catheter obstruction in the next 72 h, the decision to switch to a caudal block was made. This case demonstrates ESP block's opioid-sparing effect, improving the overall life quality of a patient. While no case reported any complications, every report points out technical and anatomical difficulties that may arise during block performance. For instance, the injection depth to the transverse process will significantly vary between patients of different ages (60). Therefore, all reports note the absolute requirement of well-trained needle puncturing skills and a stable patient position to ensure the success of the block.

#### 3.3.2 Impar ganglion block

Impar ganglion is a bundle of sympathetic nerves surrounding the sacrococcygeal joint in front of the coccyx. Blocking these nerves relieves sympathetic and visceral pains in the coccygeal and perineal areas (61). The most common approach of introducing the block is the trans-sacrococcygeal approach, in which a straight needle is inserted into the sacrococcygeal joint under sonographic guidance (62). Although effective in treating chronic intractable pain, impar ganglion blocks are rare. A single case report describing the use of impar ganglion block in a pediatric patient was found. A 3-year-old patient with refractory perineal pain caused by embryonal bladder rhabdomyosarcoma received a neurolytic injection on the impar ganglion (30). After the procedure, the patient experienced improvement on the FLACC scale with 70% pain reduction. The patient had less frequent pain breakthroughs and a better sleep pattern. However, the patient did not experience any opioid reduction and was still receiving co-adjuvants. Unfortunately, the patient died five days after the block due to tumor progression. While the block had provided some pain relief, the authors (30) note the necessity of prognosis and offer the management of the side effects after impar ganglion block, precisely bladder and rectal incontinence. Further studies in pediatric populations should be done to make a more solid conclusion about using impar ganglion block.

## 4 Special considerations: end-of-life care

The possibility of using regional blocks at the end of life as an element of palliative care deserves special consideration. Recent studies show that up to ninety percent of patients report pain, with the highest rate in cases with solid tumors (8, 63). However, pain management remains one of the weak links. In some cases, poor pain management is related to physicians not being actively involved in end-of-life care (8). Therefore, palliative care for pediatric oncological patients should include appropriate pain control (64). As already mentioned, prescribing systemic opioids is a common regimen for children with terminal cancer (65). There are obvious issues with the use of opioids, including the need to increase the dose and side effects such as nausea, vomiting, etc. Furthermore, the use of opioids in the home settings has in some cases increased risk of opioid misuse of opioids (66). In this sense, the question of the feasibility of regional analgesia in children with terminal cancer is highly relevant. Despite the limited number of publications that address continuous and single shot blocks for pain management in pediatric terminal cancer, their importance cannot be overstated (39). In a recent retrospective study, Cuviello A et al. analyzed pediatric and young adult patients who received continuous or single-shot nerve blocks as part of regional pain management at the end of life (55). The patient cohort included twenty-seven patients, of whom twenty-two (81.5%) were diagnosed with solid tumors. We summarize the data from this study, excluding young adult patients in Table 4. The authors conclude that performing regional anesthesia at this stage is not only technically feasible, but also significantly improves the patient's quality of life (55).

**Table 4:** Fifteen cases of regional blocks for children with solid tumor diagnoses at the end of life.

Age, years	Description/diagnosis	Location and type of pain	Type of block and location	Anesthetic agent(s) at insertion	Change in mean pain score at 24 h
2	Hepatoblastoma	Abdomen Nociceptive and visceral pain	Peripheral nerve block catheter, erector spinae plane block	Ropivacaine	−2
7	Osteosarcoma	Arm nociceptive pain	Peripheral nerve block catheter, Supraclavicular	Ropivacaine	4
** **		Leg	Peripheral nerve block catheter, Femoral	Ropivacaine	8
17	Granulosa cell tumor	Abdominopelvic Nociceptive and visceral pain	Intrathecal catheter, L3-L4	Ropivacaine/fentanyl	−8
16	Osteosarcoma	Arm Nociceptive pain	Peripheral nerve block catheter, Interscalene	Ropivacaine/bupivacaine	0
4	Hepatoblastoma	Chest	Epidural catheter, T6-T7	Ropivacaine	1
17	Adenocarcinoma	Lower Back Somatic pain	Intrathecal catheter, L3-L4	Bupivacaine/hydromorphone	−4
10	Smooth muscle cell tumor	Leg Neuropathic and somatic pain	Epidural catheter, L2-L3	Ropivacaine/fentanyl	−3
19	Primary central nervous system tumor	Back	Single-shot nerve block, Celiac plexus	Ethanol/bupivacaine	−6
15^a^	Neuroblastoma	Abdomen Nociceptive, visceral and somatic pain	Epidural catheter, T12-L1	Ropivacaine/fentanyl	0
15	Rhabdomyosarcoma	Perinium Nociceptive and neuropathic pain	Epidural catheter, L4-L5	Ropivacaine	−4
8	Rhabdomyosarcoma	Abdomen	Single-shot nerve block, Celiac plexus	Ethanol/Bupivacaine	−4
9^a^	Osteosarcoma	Leg Nociceptive and neuropathic pain	Epidural catheter, L4-L5	Ropivacaine/fentanyl	−10
10	Osteosarcoma	Leg Nociceptive pain	Epidural catheter, L2-L3	Bupivacaine	−2
5^a^	Rhabdomyosarcoma	Abdominopelvic Nociceptive and visceral pain	Epidural catheter, L1-L2	Ropivacaine/fentanyl	−4
3	Wilms tumor	Abdomen	Single-shot nerve block, celiac plexus	Ethanol/bupivacaine	N/A

^a^ Patients received a block for pain management for a surgical procedure or for early postoperative period.

## 5 Results

### 5.1 Risks that come with block performance and ways to resolve them

All blocks described in our study were performed either under general anesthesia (GA) or sedation. The impact the patients’ state—under GA, sedation, or awake—has on block performance remains unclear (67). Two serious adverse effects of regional anesthesia, such as local anesthetic systemic toxicity (LAST) and postoperative neurologic symptoms (PONS), are often linked to the “doubled risk” imposed by performing blocks under GA or sedation. Nevertheless, the most extensive review of over 50,000 regional anesthesia performances has shown that performing block in anesthetized children's patients is safe and should be considered standard procedure (68). According to the author, there was only one case of PONS with a small sensory deficit in a sedated patient that did not last longer than six months.

As for the LAST, there is a low incidence of such complications in children with a risk factor of 0.76:10,000 (95% CI, 0.3–1.6:10,000) (17). However, the main concern for LAST is that its initial manifestations, such as tremors, seizures, and twitching, are masked and left unnoticed when a patient is under GA or sedated. For that reason, changes in cardiac events are used to speculate LAST in anesthetized children (69). Another preventive method involves injecting test doses of local anesthetics. Even if no adverse effects were present during the test dose, the injection should be done incrementally, and aspiration should be done before each injection time. Given that LAST can occur despite all precautions, handling measures such as lipid emulsions should always be ready to prevent the situation from worsening (57).

Most pediatric cancer patients receive chemotherapy as a part of their treatment (70). The immunosuppressed state of such patients theoretically increases the possibility and severity of getting infected. While neuraxial blocks are said to decrease the risk of infection by attenuating stress response, for immunosuppressed patients, such attenuation may veil the clinical signs needed to recognize the infection (71). A delay in infection recognition can worsen neurologic complications (72). Patients receiving continuous local anesthetic infusions through catheters and pumps are also more prone to such risks. While not all collected case reports provided information about the peri- and postoperative use of antibiotics, we believe such prophylaxis was carried out since no infection-related complications were reported. Some case reports described an accidental removal or dislocation of the catheters. Such events cause subsequent replacement of the catheters, which may terminate the effect of regional anesthesia.

Inaccurate needle placement is another risk that may result in a failed procedure. As a rule, the injection for an intrathecal block should be given close to or below L4/L5 interspace level in neonates or L2/L3 in older children (51). This level difference is caused by subsequent growth and shift in the anatomic position of the spinal cord's ending. Some block types require comparatively more precision due to the significant variability of the anatomic landmarks, e.g., the narrowness of subarachnoid space in newborns is different than that of a toddler (73). The use of technology such as fluoroscopy or CT accounts for half of the block performances in our cases. Guidance techniques were described to assist the accuracy of the local anesthetic injection; meanwhile, the other half of cases were performed unassisted. While no reasoning for unassisted guidance was provided, it is for sure that unguided blocks require more skilled personnel to perform them. No complications related to an inaccurate injection and subsequent block failure were observed.

Another serious complication that can happen during block performance is a nerve damage. Although rare, nerve damage-related complications can dramatically worsen the quality of life. Any forceful intraneural injection can cause mechanical damage to the axonal structure, leading to pathophysiological changes in the nerves (74). The risks of nerve damage can be reduced using a combination of guidance techniques. Using “triple monitoring,” which includes US, nerve stimulation, and pressure monitoring, is advisable. This combination may benefit, but the extent to which it helps avoid nerve damage is poorly studied (75). It is clear, however, that using at least one type of guidance will assist in achieving higher block precision.

Nevertheless, it is essential to note that each technique has its limitations which should be handled and weighed by skilled personnel. Above, we mentioned a case of using the US to guide needle placement in a 7-months-old patient with rhabdomyosarcoma. Despite the guidance, due to presence of technical artefacts and anatomical features, anesthesiologists might not get adequate visualization, which may result in block failure or give rise to local and systemic complications following infusion of local anesthetic into incorrect or dangerous areas (76). The correct patient choice, local anesthetic, coverage area, volume, weight, and comorbidities should be strictly considered before performing a block to minimize the risks.

### 5.2 Prospective points that require further investigation

#### 5.2.1 Does parental participation in postoperative pain management affect a child's recovery?

Children placed in PICU are exposed to two simultaneously negative experiences: painful procedures and separation from parents. Such stressful and painful stimuli can interfere with their neurodevelopment and growth, inducing negative consequences to develop later in life (77). This is of particular concern in preterm infants (PTIs). Each neonate in ICU was said to undergo 8–17 procedures per day, with a mean score of painful procedures equal to 10 (78). The parental presence, which can be manifested as vocalization, skin-to-skin contact, or the odor of the mother's milk, is said to promote oxytocin system-based pain modulation (79). Existing animal studies have shown that release of oxytocin into the bloodstream after acute swim stress in rats provided an adequate analgesic effect (80). From these studies, parents’ presence and active involvement in their child's pain management can positively impact pain control in children.

Many parents often describe their willingness to be involved in caring for their hospitalized children yet need clarification about their role in a hospital setting (81). Children are thought to lack the capacity to comprehensively deliver the severity of their pain, causing most pain to be left untreated in almost half of the postoperative pediatric patients (9). According to Yang (82), as primary caregivers, the parents can deliver a better assessment of their child's pain to medical staff, thus improving the quality of pain management and building trust-based relationships between them. The evidence suggests reduced physical and emotional distress once parents can participate in managing physical care after their child (83).

However, this was only achievable when parents obtained preparatory training and information about the basic procedures performed within hospital settings, such as the meaning behind vital signs monitors or child's NIV masks (84). The evidence-based practices for parental involvement in the postoperative care of children resulted in lesser anxiety, better sleep pattern, and reduced pain scores in patients when either of the parents was present during postoperative care procedures (85). Many hospitals’ policies restrict the prolonged stay of visitors or do not allow any at all. However, if implemented wisely, both parties can benefit from improved quality of pain management and have less burden associated with untreated pain (86).

#### 5.2.2 Does ultrasound guidance help to reduce the dose of LA?

It was concluded by Willschke and his colleagues (87) that using the US as guidance in ilioinguinal and iliohypogastric nerve blocks allows the reduction of levobupivacaine to 0.075 ml/kg. This is of great importance, as using local anesthetic in big doses can lead to developing local anesthetic systemic toxicity in patients. It was also previously demonstrated that guidance reduces the risk of local anesthetic systemic toxicity (88). This suggests that there might be an optimal dose of local anesthetic for each type of block done under the guidance. More studies are needed to confirm the efficacy of all guidance types (fluoroscopy, nerve stimulator, or CT) and their relations to the dose volume.

#### 5.2.3 Should blocks be used earlier in the course of pain?

As mentioned earlier, pain becomes so intolerable at the end of life that doctors prescribe dangerous doses of opioids. While good at pain control, such pain relief comes with severe risks of developing side effects and opioid tolerance. Several cases have described patients with opioid-caused delirium that was resolved once a block was performed. We want to raise the question of whether the much earlier performance of the block can prevent such escalation of opioids and spare patients from opioid-related complications. That is not to conclude that blocks are free of risks: it is more of a need for an in-depth cost-and-benefit analysis, showing which procedure's benefit can outweigh the associated risk. No data states that performing block before turning to more severe opioid treatment yields better pain control. We understand that, depending on the patient's unique health scenario, the prescription of high-dose opioids is part of a guideline meant to deal with pain. Moreover, it is also more challenging to perform blocks on children, requiring more skilled anesthesiologists. These factors—but are not limited to them—probably contribute to the decision to use regional anesthesia techniques when conventional treatment fails. Given these circumstances, it will surely take quite a while to get the answer to the proposed question.

## 6 Conclusion

Regional anesthesia techniques can efficiently alleviate pain in different health circumstances in pediatric oncology patients. Blocks were shown to control pain after tumor resection, amputation procedure and as a part of palliative care treatment. Our case report review shows that blocks can be safely used in a diverse group of pediatric patients, with the youngest patient to receive blocks being seven months in our analysis. In most cases, the pain has already passed the point to be treated with opioids, so regional blocks were seen as the utmost pain relief measure. The prolonged pain control was achieved by tunneling catheters or inserting a pump with a local anesthetic reservoir.

All patients from reports, who received block as a part of postoperative pain control, expressed a certain level of pain relief and were able to get back to participating in life activities and spending quality time with their family and friends. We also observed the importance of psychological assistance when dealing with high pain levels, as in the case of patients who underwent amputation. This study has not examined how non-pharmacological methods were utilized for postoperative pain management. However, as seen from the report, they indeed have taken place within the settings. Among found thirty-four cases, only two cases reported bleeding and pneumothorax as complications. Although relatively safe, using regional anesthesia among children patients is a subject of many concerns, including LAST and complications that may arise during and after block performance. All cases emphasize the absolute requirement of highly skilled professionals to achieve better pain relief control.

Since this is a review of care reports, not all existing regional anesthesia techniques have been described. Therefore, a bias for the efficiency of described methods may arise. Since some case reports do not inform on the weight of the patients, this review had to neglect the dose of local anesthetic used in each case. This has limited the study's ability to address the issues related to the threshold and optimal amounts of local anesthetic for patients of different ages. Further studies on other techniques and case report collection should be done to make a sound conclusion about using regional anesthesia in childhood cancer patients.

## Data Availability

The original contributions presented in the study are included in the article/Supplementary Material, further inquiries can be directed to the corresponding author.
